# Multidisciplinary management of acute cholecystitis during the COVID-19 pandemic

**DOI:** 10.1038/s41598-023-43555-3

**Published:** 2023-09-27

**Authors:** I. Tóth, S. Ábrahám, Z. Karamya, R. Benkő, M. Matuz, A. Nagy, D. Váczi, A. Négyessy, B. Czakó, D. Illés, M. Tajti, E. Ivány, G. Lázár, László Czakó

**Affiliations:** 1 Department of Surgery, University of Szeged, Szeged, Hungary; 2 Divison of Gastroenterology, Department of Medicine, University of Szeged, Kálvária Sgt. 57., Szeged, 6725 Hungary; 3 Department of Clinical Pharmacology, University of Szeged, Szeged, Hungary; 4 Department of Radiology, University of Szeged, Szeged, Hungary; 5 Department of Urology, University of Szeged, Szeged, Hungary

**Keywords:** Diseases, Medical research

## Abstract

The coronavirus disease 2019 pandemic had a major impact on most medical services. Our aim was to assess the outcome of acute cholecystitis during the nationwide lockdown period. All patients admitted to our emergency department for AC were analysed. Patient characteristics, performance status, AC severity, treatment modality and outcome of AC were assessed during the lockdown period (Period II: 1 April 2020–30 November 2021) and compared to a historical control period (Period I: 1 May 2017–31 December 2018). AC admissions increased by 72.8% in Period II. Patients were younger (70 vs. 74 years, p = 0.017) and greater in number in the CCI 1 group (20.4% vs. 11.2%, p = 0.043) in Period II. The unplanned readmission rate (6.3 vs. 0%, p = 0.004) and the gallbladder perforation (GP) rate was higher (18.0 vs. 7.3%, p = 0.006) in Period II. Percutaneous transhepatic gallbladder drainage (PTGBD) was more frequent (24.1 vs. 12.8%, p = 0.012) in Period II. In addition to a drop in patient age and CCI, a significant rise in the prevalence of acute cholecystitis, GP and unplanned readmissions was observed during the nationwide lockdown due to the COVID-19 pandemic. PTGBD was more frequent during this period, whereas successful conservative treatment was less frequent.

## Introduction

The emergence of the novel coronavirus (SARS-CoV-2—severe acute respiratory syndrome coronavirus 2) and the COVID-19 (coronavirus-2019) pandemic it caused transformed healthcare considerably all over the world. In early 2020, the development of the epidemic (and its later growth into a pandemic) resulted in important epidemiological measures taken in all countries. In Hungary, in early March 2020, healthcare was “locked down” with a focus on providing care for COVID-19 patients and controlling the spread of the epidemic. Apart from halting or delaying non-urgent surgical procedures, there were significant changes in the approach to managing emergency conditions from a surgical perspective^1^. As elective surgeries, including elective cholecystectomies, were put on hold by government directive, the management of acute cholecystitis (AC), a common emergency condition, also changed during the pandemic^2^. In most centres, the management of AC is based on the Tokyo Guidelines published in 2013 and later amended in 2018^3,4^, the integral parts of which are, depending on the severity of AC, conservative therapy, surgical treatment (that is, cholecystectomy) and percutaneous transhepatic gallbladder drainage (PTGBD).

The objective of our study was to analyse the changes in the management of AC at the University of Szeged during the COVID pandemic.

## Patients and methods

Data from patients diagnosed with AC who had received care at the University of Szeged in the pre-COVID period (Period I: from 1 May 2017 to 31 December 2018, 20 months) and during the COVID period (Period II: from 1 April 2020 to 30 November 2021, 20 months) were evaluated retrospectively. In addition to gender, age, mortality data and readmissions, the current general condition of the patients was also determined. To this end, the Charlson Comorbidity Index (CCI) was used, which predicts 10-year survival taking comorbidities and patient age into account^5^. Three groups were formed using CCI (Group 1: 0 points; Group 2: 1–3 points; Group 3: 4–10 points). During Period II, patients were routinely screened with a SARS-CoV-2 PCR (polymerase chain reaction) test. Based on aspects specified in the 2018 Tokyo Guidelines (TG18/TG13 severity grading for acute cholecystitis^6^), AC cases were classified into three groups of severity (Grade I [mild], Grade II [moderate] and Grade III [severe]). Based on an abdominal ultrasound (US) scan, AC cases were classified according to several morphological diagnoses: simple acute calculous cholecystitis, empyema vesicae felleae (EVF), gallbladder perforation (GP—confirmed by computed tomography) and hydrops vesicae felleae (HVF). Based on our radiological standards, acute calculous cholecystitis has general US signs as sensitive findings (sonographic Murphy sign, presence of cholelithiasis) and less specific findings (gallbladder wall thickening (over 3 mm), sludge, increased vascularisation of the gallbladder wall, pericholecystic fluid, gallbladder distension, layering of the gallbladder wall). In case of EVF additional to general signs of calculous cholecystitis can be seen: echogenic content within the gallbladder lumen. In case of GP the following signs can be observed: defect in the gallbladder wall with pericholecystic fluid collection, stranding of the omentum, adjacent hepatic abscess. In case of HVF you can see impacted stone in cystic duct, > 4 cm transverse diameter of gallbladder, > 9 cm longitudinal diameter of gallbladder and convex borders of gallbladder. Patients under 18 years, cases with acalculous cholecystitis or accompanying acute pancreatitis were excluded.

**Figure 1:**
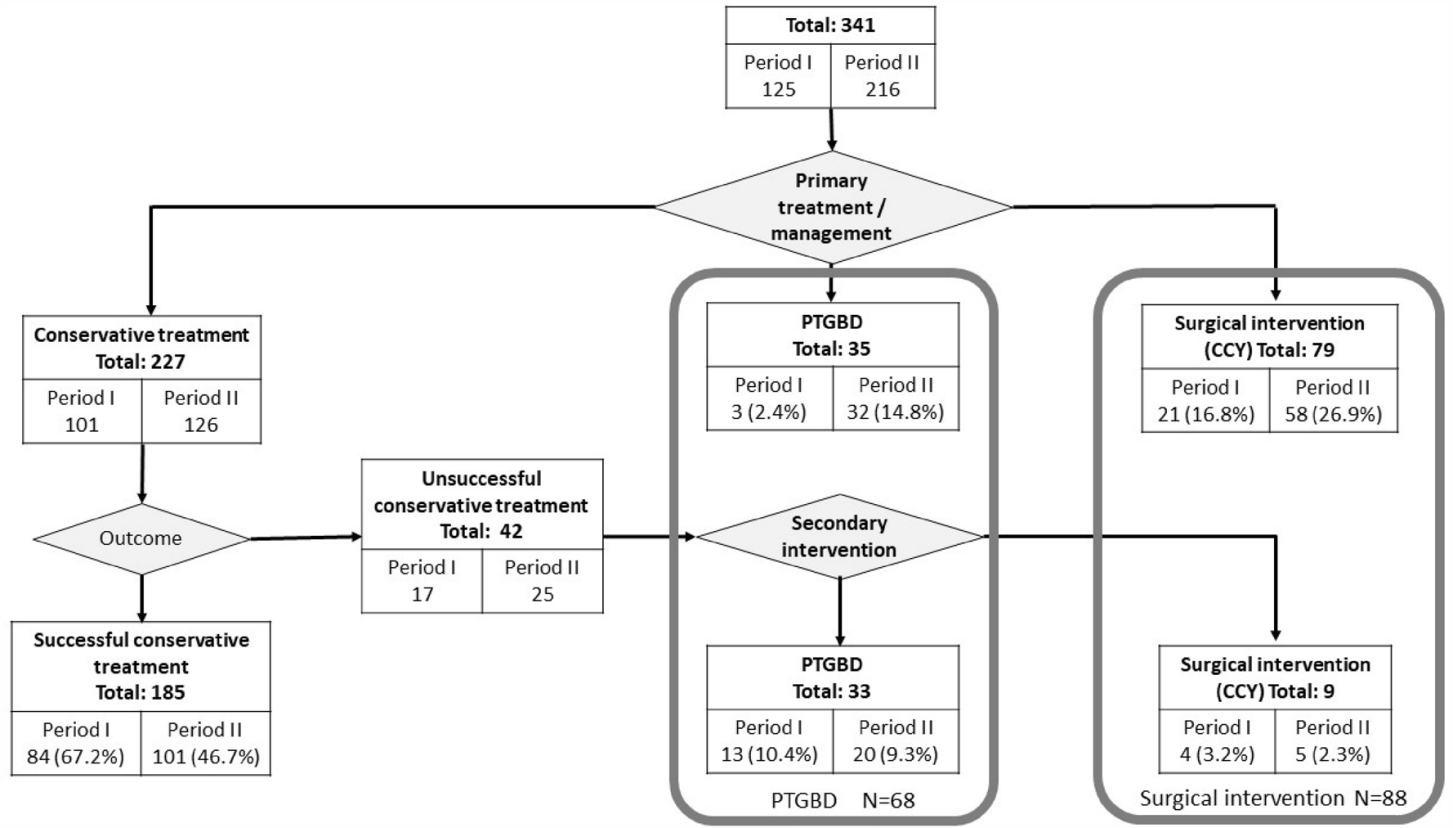
Flowchart for the management and number of patients in each group (*PTGBD* percutaneous transhepatic gallbladder drainage, *CCY* cholecystectomy).

Multidisciplinary management encompasses three alternative treatment methods in the management of AC. The first is conservative medical therapy, the second is a surgical procedure (cholecystectomy, CCY), and the third is PTGBD. If conservative therapy was used first but failed, either surgery or PTGBD can be considered as secondary intervention, depending on the circumstances (time frame, severity of AC, general condition of the patients or CCI). See Fig. 1 for treatment pathways.

Surgical treatment was assessed by type of surgery performed (laparoscopic cholecystectomy [LC], converted LC or primary open surgery), while conversion rate (CR = number of converted LCs × 100/[total number of surgeries − number of primary open cholecystectomies]) and laparoscopic success rate (LSR = number of LCs/total number of surgeries) were evaluated as measures of surgical efficacy. The epidemiology, severity of AC (CCI, grade and ultrasound morphological diagnoses), multidisciplinary management pathways and outcome of the treatment (mortality or readmission) were compared in the cohorts in the two periods.

Welch’s test, one-way ANOVA, Pearson’s chi-squared test, Fisher’s exact test and Independent-Samples Mann–Whitney U Test were used to statistically analyse patient characteristics, surgical treatments, length of hospital stay, mortalities and unexpected readmissions. CCI groups, ultrasound morphological groups, AC severity groups and treatments were analysed statistically using Chi-Square Test with Pairwise Z-Tests with Bonferroni correction.

All methods were carried out in accordance with relevant guidelines and regulations. Informed consent was obtained from all subjects. The study was approved by the Regional Human Biomedical Research Ethics Committee at the University of Szeged (81/2020-SZTE).

## Results

**Figure 2:**
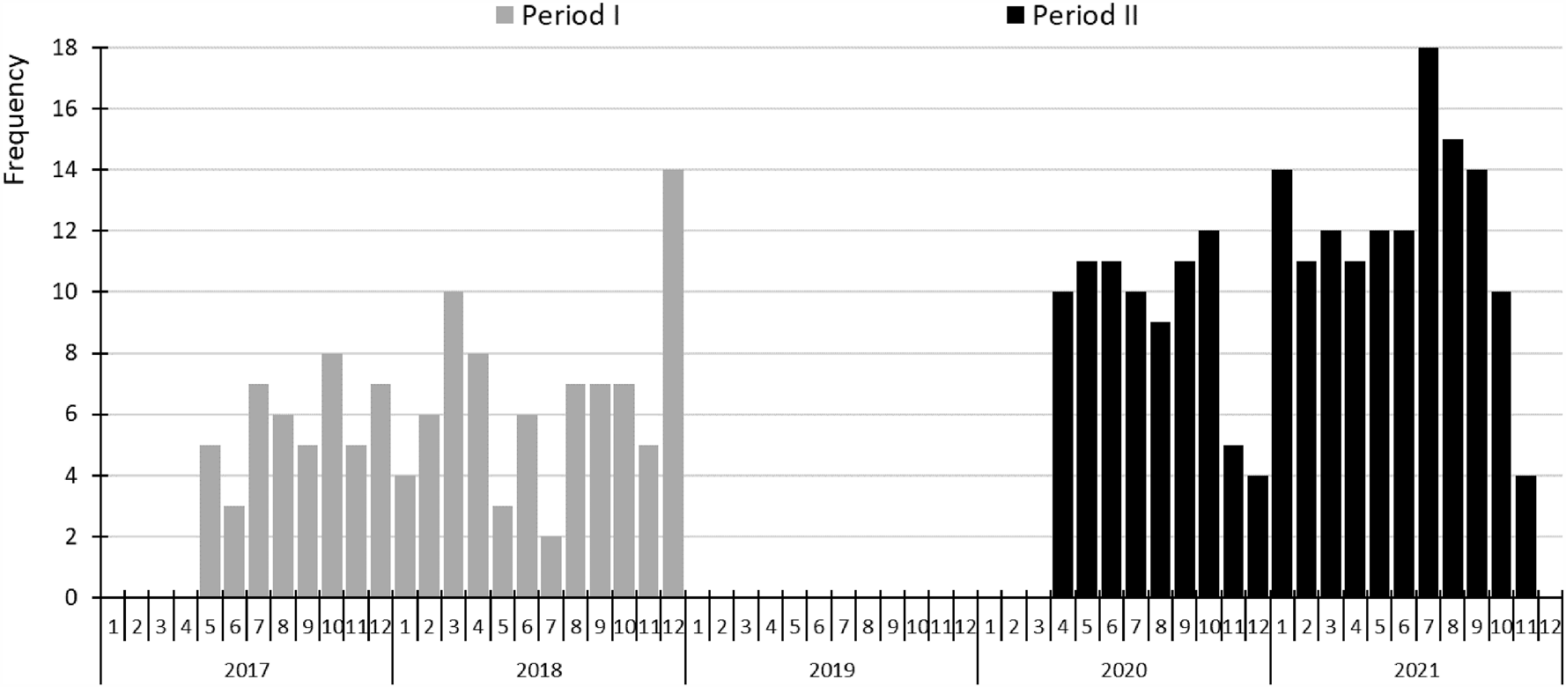
Number of acute cholecystitis diagnoses per month in the two periods.

A total of 341 patients received care for AC at the University of Szeged during the study periods. There were 125 patients in Period I and 216 in Period II, a significant increase of 72.8% (p < 0.001) (Fig. 2).

Out of the 216 patients, only six (2.8%) tested positive for COVID. The median age of the patients was significantly lower in Period II (70 vs. 74 years, p = 0.017). The gender ratio did not change, with a predominance of females (56 vs. 56.5%, p = 0.51). As for CCI classification, the rate of cases classified into CCI Group 1 was significantly higher in Period II (20.4 vs. 11.2%, p = 0.043) (Table 1).

**Table 1:** General patient and cholecystitis characteristics.

	Period I (pre-COVID)	Period II (COVID)	p value
N	125 (100%)	216 (100%)	< 0.001
Age			
Median (min–max)	74 (27–101)	70 (24–96)	0.017
Sex			
Female	70 (56%)	122 (56.5%)	NS
Male	55 (44%)	94 (43.5%)	
CCI scores			
Group 1 (0)	12 (11.2%)	40 (20.4%)	0.043
Group 2 (1–3)	36 (33.6%)	57 (29.1%)	NS
Group 3 (4–10)	59 (55.1%)	99 (50.5%)	NS
NA	18	20	
AC morphological diagnosis (US)			
Calculous cholecystitis	77 (62.6%)	139 (64.4%)	NS
Empyema vesicae felleae	4 (3.2%)	3 (1.4%)	NS
Gallbladder perforation	9 (7.3%)	39 (18.1%)	0.006
Hydrops vesicae felleae	33 (26.8%)	35 (16.2%)	0.019
NA	2		
AC severity (TG13/18)			
Grade I	49 (39.2%)	75 (34.7%)	NS
Grade II	66 (52.8%)	119 (55.1%)	NS
Grade III	10 (8%)	22 (10.2%)	NS

*AC* acute cholecystitis, *CCI* Charlson comorbidity index, *COVID* coronavirus disease, *NA* no data, *TG13/18* Tokyo Guidelines 2013/2018, *US* ultrasound, *NS* not significant.

There was no significant change in the severity of AC, with the rate of Grade II cases being the highest in both groups (55.1 vs. 52.8%). As regards ultrasound morphological diagnoses, the GP rate rose significantly (18.1 vs. 7.3%, p = 0.006) in Period II and that of HVF fell significantly (16.8 vs. 26.8%, p = 0.019) in the same period (Table 1). There was significant difference between the two periods in the length of hospital stay, median hospital stay in Period II was shorter by 1 day (8 vs. 7 days, p = 0.011) (Table 2). There was no significant difference in mortality either during the hospital stay or within 30 days after the procedure (Table 2). As regards unplanned readmission within 30 days, significant differences were observed. While there were no such incidents during Period I, twelve cases required readmission during Period II (0 vs. 6.3%, p = 0.004) (Table 2).

**Table 2:** Treatments and perioperative data.

	Period I (pre-COVID)	Period II (COVID)	p value
N			
Treatment/management	125	216	
Successful conservative treatment	84 (67.2%)	101 (46.8%)	< 0.001
Percutaneous drainage	16 (12.8%)	52 (24.1%)	0.012
Primary + secondary	3 + 13	32 + 20	
Surgical treatment	25 (20%)	63 (29.2%)	NS
Primary + secondary	21 + 4	58 + 5	
Surgical treatment			
Laparoscopic cholecystectomy	19 (76%)	49 (77.8%)	NS
Converted laparoscopic cholecystectomy	4 (16%)	13 (20.6%)	
Open cholecystectomy	2 (8%)	1 (1.6%)	
CR (%)	17.4	20.9	NS
LSR (%)	76	77.78	NS
Length of hospital stay			
N	117	201	
NA	8	15	
Median (min–max)	8 (1–62)	7 (1–60)	0.011
Mortality			
No	116 (95.08%)	198 (94.29%)	NS
Yes	6 (4.92%)	12 (5.71%)	
NA	3	6	
30-day mortality			
No	113 (98.26%)	192 (96.97%)	NS
Yes	2 (1.74%)	6 (3.03%)	
NA	10	18	
Unplanned readmission			
No	115 (100%)	177 (93.65%)	0.004
Yes	0 (0%)	12 (6.35%)	
NA	10	27	

*CCY* cholecystectomy, *COVID* coronavirus disease, *CR* conversion rate (number of converted laparoscopic cholecystectomies × 100/[total number of surgeries − number of primary open cholecystectomies]), *LSR* laparoscopic success rate (number of laparoscopic cholecystectomies/total number of surgeries), *NA* no data.

**Figure 3:**
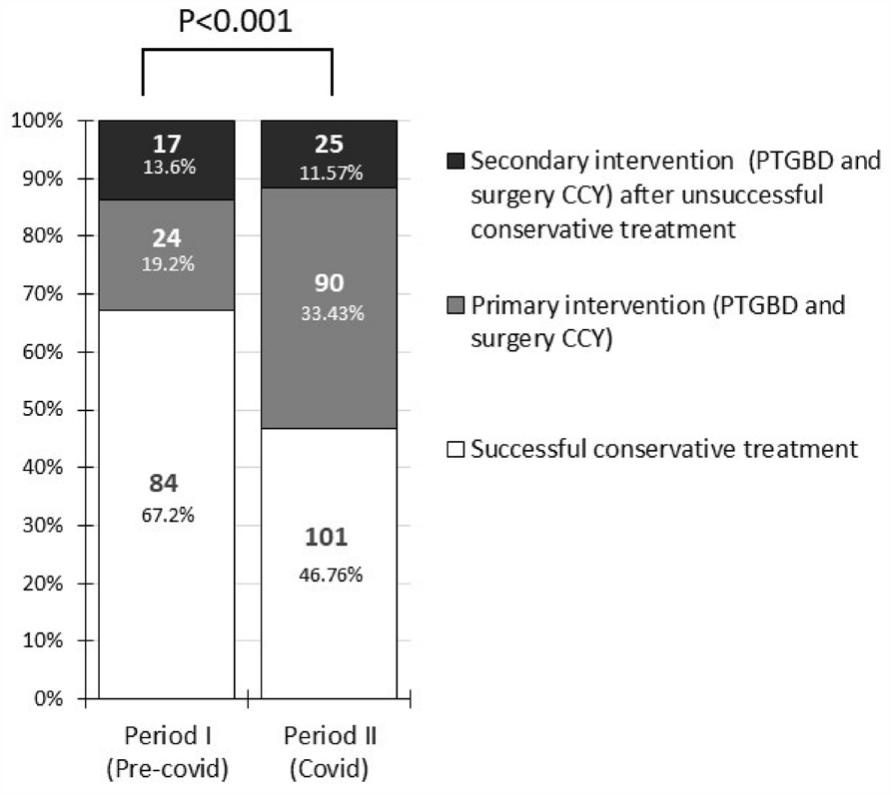
The distribution of treatment types in the two periods (*CCY* cholecystectomy, *COVID* coronavirus disease, *PTGBD* percutaneous transhepatic gallbladder drainage).

There was a significant change in the rates of the treatment methods between the two periods (Fig. 3). In Period I, successful conservative therapy demonstrated a significantly higher rate (67.2 vs. 46.8%, p < 0.001), whereas the rate of total PTGBD only showed a marked increase (24.1 vs. 12.8%, p = 0.012) in Period II, with no significant change in the surgery rate. Out of the six COVID-positive patients, two received successful conservative therapy, three underwent PTGBD, and one had a converted LC.

When assessing (both primary and secondary) surgeries, we found no significant difference either in the distribution of surgery types (LC, converted LC and primary open surgery) or in CR (17.4 vs. 20.9%) and LSR (76 vs. 77.8%) between the two periods.

## Discussion

This study demonstrated a significant rise in the incidence of AC, a drop in patient age and CCI, and significant growth in the prevalence of GP, unplanned readmission and the rate of PTGBD during the nationwide lockdown due to the COVID-19 pandemic.

The epidemiological measures taken during the COVID-19 pandemic have transformed the structure and conditions of healthcare considerably. Several medical departments were either closed down or designated as COVID care facilities, resulting in a significant number of clinicians having to provide care for COVID patients. With regard to surgical care, non-emergency procedures, such as elective cholecystectomies, were immediately suspended in compliance with the lockdown measures.

Considering these circumstances, it is not surprising that the number of patients with AC increased substantially in Period II, since patients with gallstones were only treated if acute inflammation was also present and elective cholecystectomies were suspended. An Irish study reported similar findings on the number of AC cases, and it even supposed that a possible reason for this was an excessive consumption of fatty food based on the “stay-at-home” principle^7^.

A study examining elective cholecystectomies from the United Kingdom showed that in the pre-pandemic group a higher proportion of operations were performed for non-inflammatory pathology compared to the post-COVID recovery phase^8^. There was a notable contrast observed in Period II, as the median age of patients receiving care was significantly lower compared to the previous period. Younger patients who had usually undergone surgery with milder symptoms before an acute inflammatory event in Period I required care for AC during the pandemic.

During COVID, healthcare capacities dropped, with every level of the healthcare provision system from general practitioners to tertiary centres focussing on COVID. Patients frequently sought medical attention or accessed suitable healthcare providers after experiencing symptoms for several days and reaching an advanced stage of inflammation^9^. This was well indicated by the significant change in the rates of morphological diagnoses made based on the ultrasound scan. In Period II, the GP rate rose considerably compared to Period I, clearly due to late treatment and lack of elective management. A study conducted at a German tertiary centre yielded a similar result, though the elevated GP rate was characteristic of the older patients under investigation^10^.

A significant change was observed in the composition of multidisciplinary management. In Period II, the PTGBD rate was significantly higher, successful conservative therapy showed a significantly lower rate, and there was no significant change in the surgery rate. A systematic review yielded comparable findings; however, the study reported that while the PTGBD rate was higher during the COVID period, there was also a higher rate of conservative therapy and a decrease in the rate of surgical treatment^11^. A recent article from July 2023 found similar results by examining data from US Academic Centers (comparing 15–15 months pre-pandemic and pandemic periods)^12^. The priority given to drainage indicates that in more advanced cases (Grade III AC, high CCI, and bad general condition of the patients), surgery is no longer the first-line option, conservative therapy alone is no longer sufficient, and therefore drainage is the best option. An Italian study clearly recommends PTGBD as primary therapy for COVID-positive patients and even for non-COVID patients for whom conservative therapy has failed or who are not fit for surgery^13^. However, in analysing data from the pre-COVID period and the first two COVID waves, a British study found no difference in the success rate for conservative therapy^14^. In addition, it should be noted that as regards surgical therapy at our department (either primary or secondary), CR and LSR were similar in both periods and the rate of laparoscopic procedures did not fall during the pandemic despite the fact that we faced more difficult cases. Laparoscopic cholecystectomy was safely used during the pandemic, as also demonstrated in a large number of cohorts^15^.

The median length of hospital stay exhibits significant difference. The implementation of minimal doctor-patient contact and reduced capacity during the COVID pandemic might have accounted for the 1-day earlier discharge of patients in Period II. The high rate of unexpected readmissions during Period II may have been caused by the higher GP rate in addition to the 1-day shorter hospital stay.

Due to the limited number of cases, the mortality data does not allow for any conclusive inferences to be made; larger studies are therefore needed.

## Conclusion

Our data suggests that imposing a restriction on elective cholecystectomies can lead to an increased occurrence of acute cholecystitis in younger patients with fewer underlying conditions. However, such patients may experience a higher frequency of perforation and readmissions. As a result, PTGBD may emerge as a more significant treatment option, alongside acute cholecystectomy, for managing AC in a comprehensive manner, particularly when conservative therapy is less successful.

## Data Availability

The data presented in this study are available on request from the corresponding author.

## Abbreviations

AC: Acute cholecystitis
COVID-19: Coronavirus disease 2019
SARS-CoV-2: Severe acute respiratory syndrome coronavirus 2
CCI: Charlson Comorbidity Index
PCR: Polymerase chain reaction
GP: Gallbladder perforation
PTGBD: Percutaneous transhepatic gallbladder drainage
US: Abdominal ultrasound
GP: Gallbladder perforation
EVF: Empyema vesicae felleae
HVF: Hydrops vesicae felleae
CCY: Cholecystectomy
LC: Laparoscopic cholecystectomy
CR: Conversion rate
LSR: Laparoscopic success rate
